# Treadmill exercise can regulate the redox balance in the livers of APP/PS1 mice and reduce LPS accumulation in their brains through the gut-liver-kupffer cell axis

**DOI:** 10.18632/aging.205432

**Published:** 2024-01-29

**Authors:** Shunling Yuan, Yirong Wang, Jialun Yang, Yingzhe Tang, Weijia Wu, Xiangyuan Meng, Ye Jian, Yong Lei, Yang Liu, Changfa Tang, Zhe Zhao, Fei Zhao, Wenfeng Liu

**Affiliations:** 1 Hunan Provincial Key Laboratory of Physical Fitness and Sports Rehabilitation, Hunan Normal University, Changsha 410012, China; 2 Hunan Sports Vocational College, Changsha 410019, China; 3 Changsha Hospital of Traditional Chinese Medicine (Changsha Eighth Hospital), Changsha 410199, China

**Keywords:** Alzheimer’s disease, treadmill exercise, hepatic oxidative stress, intestinal-liver axis, Kupffer cells

## Abstract

A growing body of clinical data has shown that patients with Alzheimer’s disease (AD) have symptoms such as liver dysfunction and microbial–gut–brain axis dysfunction in addition to brain pathology, presenting a systemic multisystemic pathogenesis. Considering the systemic benefits of exercise, here, we first observed the effects of long-term treadmill exercise on liver injuries in APP/PS1 transgenic AD mice and explored the potential mechanisms of the gut–liver–brain axis’s role in mediating exercise’s ability to reduce bacterial lipopolysaccharide (LPS) pathology in the brain. The results showed that the livers of the AD mice were in states of oxidative stress, while the mice after long-term treadmill exercise showed alleviation of their oxidative stress, their intestinal barriers were protected, and the ability of their Kupffer cells to hydrolyze LPS was improved, in addition to the accumulation of LPS in their brains being reduced. Notably, the livers of the AD mice were in immunosuppressed states, with lower pro-oxidative and antioxidative levels than the livers of the wild-type mice, while exercise increased both their oxidative and antioxidative levels. These results suggest that long-term exercise modulates hepatic redox homeostasis in AD mice, attenuates oxidative damage, and reduces the accumulation of LPS in the brain through the combined action of the intestine–liver–Kupffer cells.

## INTRODUCTION

Alzheimer’s disease (AD), commonly known as dementia or geriatric cognitive impairment, is a widely prevalent neurodegenerative disease with clinical manifestations of progressive memory impairment, cognitive dysfunction, personality changes, language impairment, and other neuropsychiatric symptoms that are currently difficult to cure [1–3]. Epidemiological surveys have shown that the number of existing cases of dementia worldwide had exceeded 51 million in 2019, and it has been estimated to reach approximately 66 million in 2030 and 115 million in 2050 [4, 5]. In the case of progressive and significant aging, AD has become a major disease affecting human health.

The classic pathology of an AD brain is characterized by neurogenic fiber tangles caused by abnormal aggregates of amyloid β-protein (Aβ) plaques and hyperphosphorylated tau (phosphorylated tau (p-tau)), as well as microglia hyperactivation, neuronal loss, and synaptic dysfunction [6, 7]. However, the current pharmacological treatments developed to tackle the pathological features of the brain have successively failed. Kwangsik Nho et al. measured the markers of liver function in serum and the cognitive performance and AD pathological features of 1174 AD patients and 407 normal elderly people, and they found a strong correlation between AD diagnosis and an increased ratio of alanine transaminase (ALT) to aspartate transaminase (AST), both of which are markers of liver function in serum [8]. An increasing number of studies have found gut microbial dysbiosis in AD patients [9–11]. Zhao et al. found that bacterial lipopolysaccharides (LPS) were present in the brain lysates from the hippocampus and superior temporal lobe neocortex in the brains of AD patients, and that the LPS levels were 26-fold higher in the hippocampal brain lysates from patients with advanced AD compared to the age-matched controls [12]. It has been suggested that there is a peripheral pathogenesis of AD which may affect brain pathology through the microbial–gut–liver–brain axis, and interventions from these points may be an effective way to treat AD.

When the AD intestinal barrier is compromised, LPS and intestinal bacteria are more likely to enter the bloodstream and then enter the liver through the portal vein to be phagocytosed and detoxified in order to avoid excess flow into the bloodstream and, subsequently, systemic inflammation and brain damage [13, 14]. When pathogenic microorganisms and LPS come in contact with Kupffer cells upon entering the liver, it will trigger an immune response and a burst of reactive oxygen respiration that may lead to inflammation and oxidative damage [15, 16]. When the function of the liver is impaired, it may further lead to a disruption of the blood–brain barrier, a “brain leak”, and a decrease in the ability of the liver to clear Aβ, thus exacerbating the pathology of the brain [17]. Unfortunately, experimental studies on the guts and livers of AD patients are very limited. AD exhibits systemic morbidity, and interestingly, appropriate physical exercise has systemic benefits for the body based on which exercise intervention has become one of the effective means to improve AD [18, 19]. The ameliorative effects of exercise on the pathology of the brain in AD patients have been widely demonstrated, but the effects on peripheral organs, such as the liver, have rarely been reported in the literature. In our study, we employed 3-month-old APPswe/PS1dE9 (APP/PS1) mice, which at this stage lack β-amyloid plaques. By six to seven months, they display Aβ deposition and memory deficits [20]. This makes them suitable for simulating key pathological features of AD and exploring early AD interventions. In this study, we determined whether there was liver damage in an APP/PS1 transgenic AD mouse model, and we observed the effects of treadmill exercise on hepatic oxidative stress and the intestinal–Kupffer cell axes in AD mice and explored the peripheral-central mechanism of exercise for improving AD in order to provide a new perspective and experimental basis for the study of AD.

## RESULTS

### The AD mice had liver damage, which was alleviated after treadmill exercise

**Figure 1:**
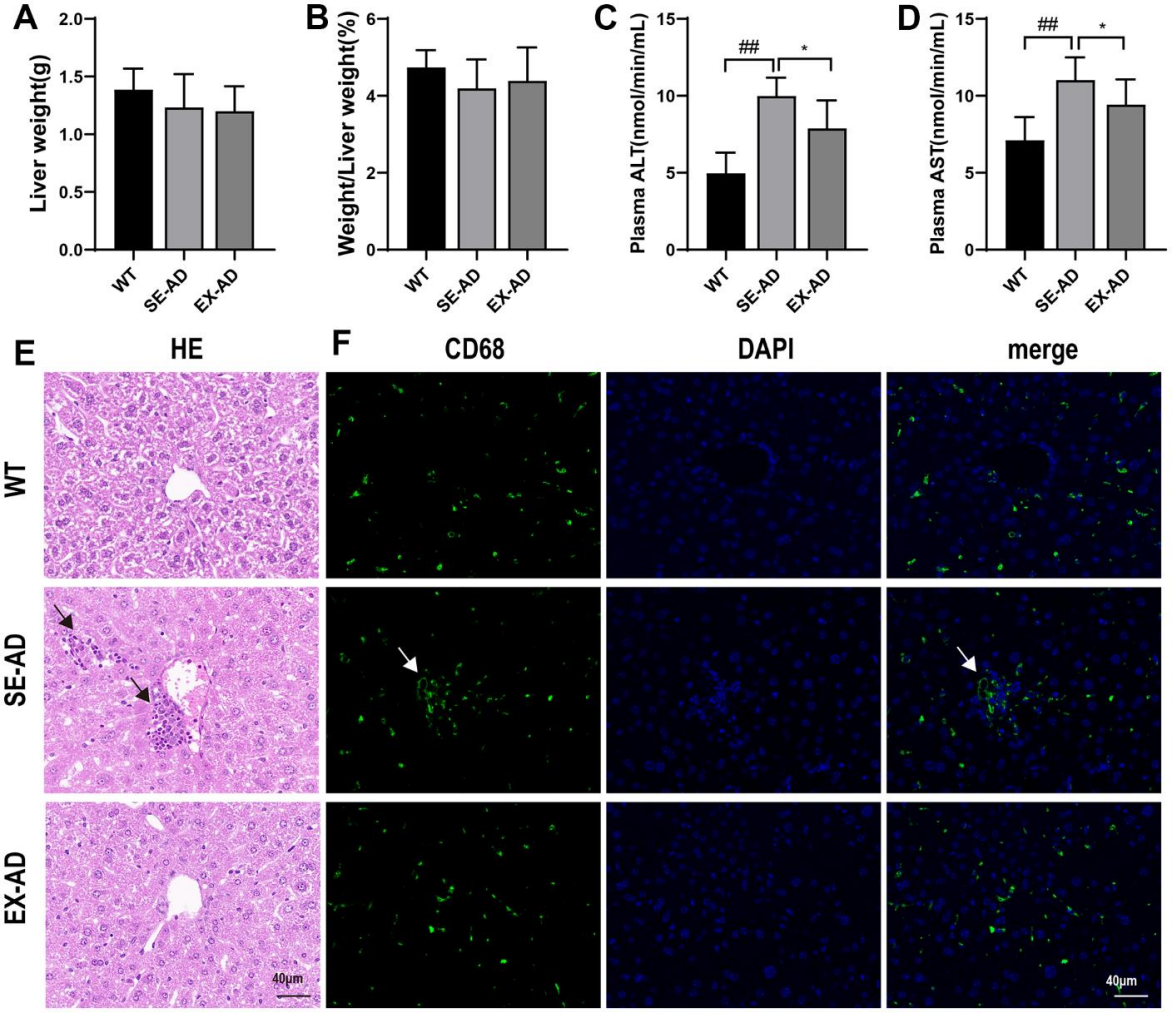
**AD mice with liver damage that was alleviated after exercise.** (**A**) Weights of the mouse livers (n = 8 per group). (**B**) Ratios of the mouse livers to body weights (n = 8 per group). (**C**) Serum glutathione aminotransferase contents (n = 6 in each group). (**D**) Serum glutathione aminotransferase contents (n = 6 in each group). (**E**) Representative images of the liver HE staining, with the black arrows indicating the abnormally aggregated Kupffer cells (n = 4 in each group). (**F**) Representative images of the liver CD68 immunofluorescence staining, with the white arrows indicating the abnormally aggregated Kupffer cells (n = 4 in each group). The data are expressed as means ± SDs. ## p < 0.01 compared with the WT group and * p < 0.05 compared with the SE-AD group.

As shown in Figure 1, the liver weights (Figure 1A) and liver-to-body weight ratios (Figure 1B) were lower in the AD mice than in the normal mice, but no significant changes were observed after exercise. Serum AST and ALT are markers of liver injury [21], and the significant increase in serum AST and ALT in the AD mice and their significant decrease after exercise (Figure 1C, 1D) suggested that liver injury may have existed in the AD mice, and it was alleviated after participation in treadmill exercise. The hepatocytes of the WT mice were arranged more regularly and were clearly spaced, and the hepatic sinusoids were distributed among the hepatocytes in greater numbers and arranged in a radioactive pattern. The hepatocytes of the AD mice were arranged sparsely and the intercellular boundaries were not obvious, and the hepatic sinusoids were fewer and irregular, as shown by the black arrows in the figure, with a large number of Kupffer cells or neutrophil aggregates. In contrast, the number of hepatocytes was significantly increased in the AD mice after exercise, and the hepatic sinusoids were increased and tended to be radiologically aligned, the cell boundaries were improved, and Kupffer cells or neutrophils were scattered among the hepatocytes (Figure 1E). Further, CD68 immunofluorescence was used to label the Kupffer cells [22], as indicated by the white arrows in the figure, and it was determined that the abnormal aggregation in the HE staining was Kupffer cells (Figure 1F). These data suggested that the AD mice had liver damage that was alleviated by exercise.

### Treadmill exercise reduced hepatic oxidative stress in the AD mice

**Figure 2:**
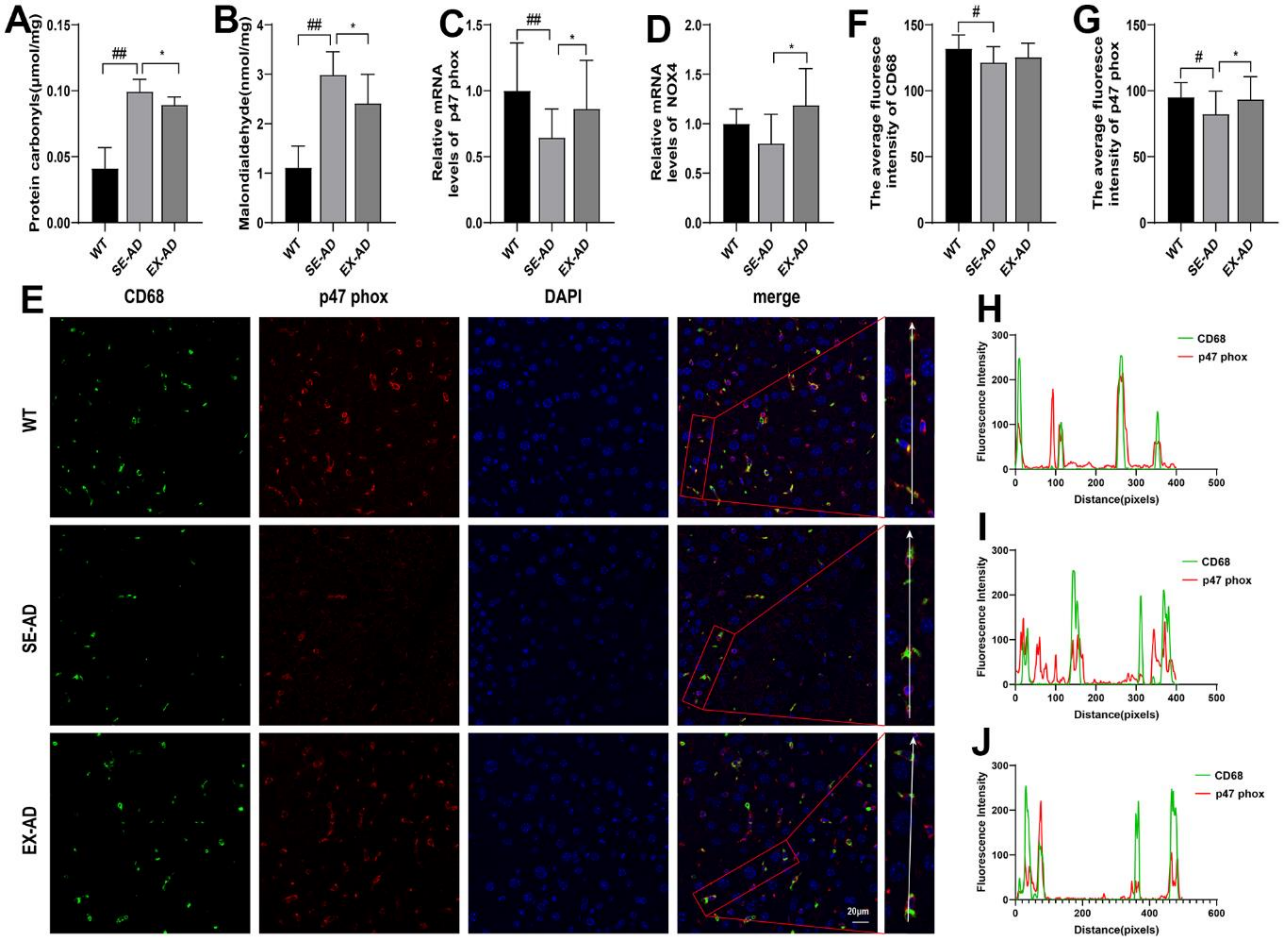
**Exercise attenuated the oxidative damage in the livers of the AD mice.** (**A**) Protein carbonyl contents in the livers (n = 6 per group). (**B**) MDA contents in the livers (n = 6 per group). (**C**) Relative expression of p47 phox mRNA in the livers (n = 6 per group). (**D**) Relative expression of NOX4 mRNA in the livers (n = 6 per group). (**E**) CD68 and p47 phox immunofluorescence staining representative images of the livers (n = 4 per group). (**F**) Quantitative statistics of the mean fluorescence intensity of CD68. (**G**) Quantitative statistics of the mean fluorescence intensity of p47 phox. (**H**) Fluorescence intensity changes in CD68 and p47 phox in the WT group. (**I**) Fluorescence intensity changes in CD68 and p47 phox in the SE-AD group. (**J**) Fluorescence intensity changes in CD68 and p47 phox in the EX-AD group. The data are expressed as means ± SDs. # p < 0.05 and ## p < 0.01 compared with the WT group, and * p < 0.05 compared with the SE-AD group.

Kupffer cells in the liver may undergo a respiratory burst when exposed to pathogenic microorganisms from the gut or LPS, and the excessive production of reactive oxygen species may lead to oxidative stress. We examined liver oxidative stress in mice, and as shown in Figure 2, the liver protein carbonyl and MDA contents were significantly increased in the AD mice and they significantly decreased after exercise (Figure 2A, 2B). This indicated that oxidative damage was present in the livers of the AD mice, and it was alleviated after exercise. Interestingly, the prooxidant genes were further examined and it was found that the expressions of prooxidant genes were low in the livers of the AD mice, and exercise instead increased the expression of prooxidant genes. To explore the relationship between Kupffer cells and oxidative damage, CD68 was co-localized with the p47 phox prooxidant protein by immunofluorescence, and it was found that CD68 expression was lower in the AD mice compared to the wild-type mice, the p47 phox protein was consistent with the gene assay, and the CD68 and p47 phox fluorescence intensity changes were consistent in all three groups of mice. The above results suggested that oxidative stress existed in the livers of the AD mice, and it was alleviated after exercise. The results also suggested that Kupffer cells are involved in the regulation of oxidative damage in the liver.

### Treadmill exercise could regulate GSH in the livers of the AD mice to enhance antioxidant capacity

**Figure 3:**
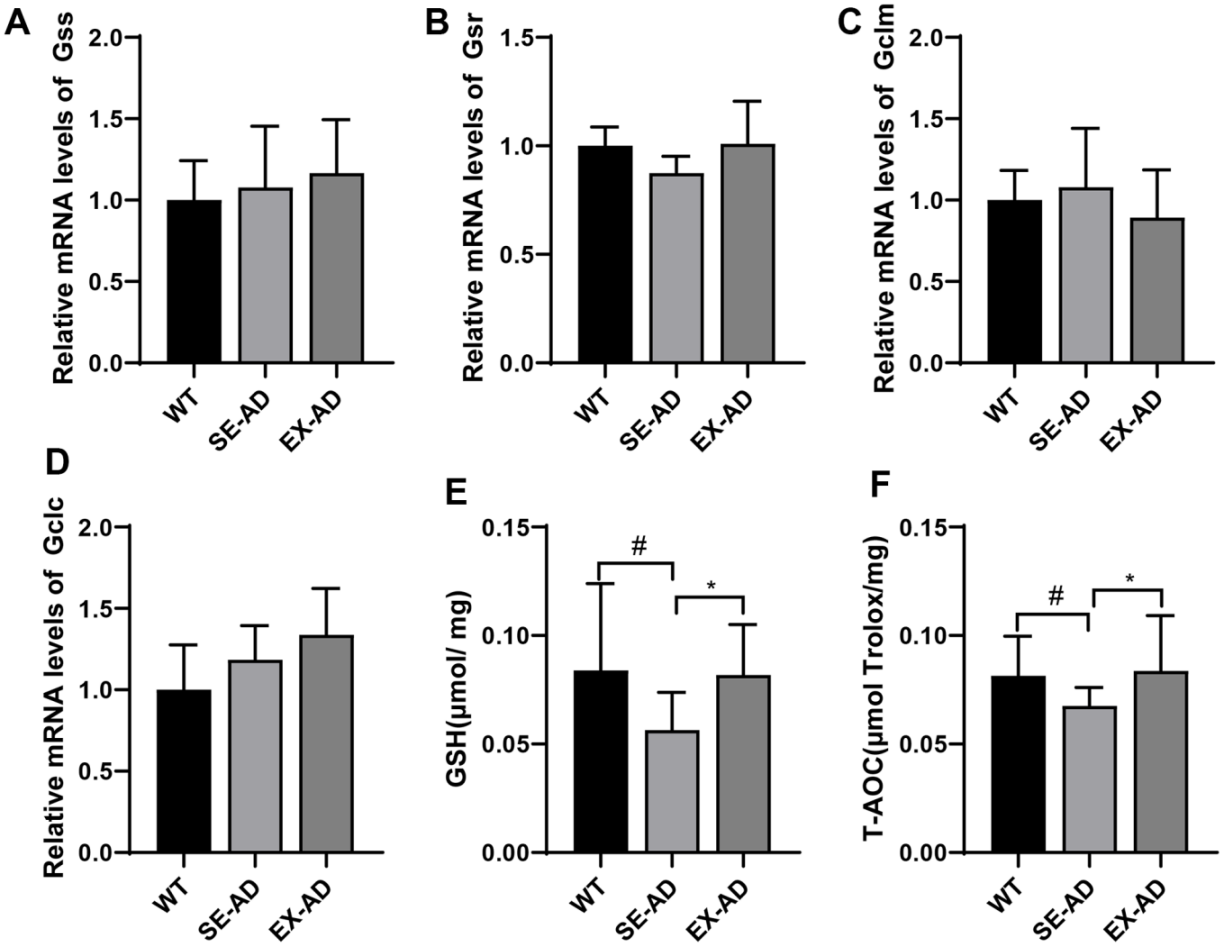
**Glutathione system and total antioxidant capacities of the animals’ livers.** (**A**) Relative expressions of Gss mRNA. (**B**) Relative expressions of Gsr mRNA. (**C**) Relative expressions of Gclm mRNA. (**D**) Relative expressions of Gclc mRNA. (**E**) Contents of GSH in the animals’ livers. (**F**) Total antioxidant capacities of the animals’ livers (n = 6 in each group). The data are expressed as means ± SDs. # p < 0.05 compared with the WT group and * p < 0.05 compared with SE-AD group.

The levels of prooxidant were low in the livers of the AD mice, but oxidative damage was present, suggesting a possible defect in antioxidant capacity. The genes controlling the production of GSH (Gss, Gsr, Gclc, and Gclm) were examined and there were no significant differences in the changes between them, but exercise appeared to have a tendency to increase the expressions of Gss, Gsr, and Gclc (Figure 3A, 3B, 3D). The GSH contents and total antioxidant capacities in the livers of the AD mice were significantly lower than those of the wild-type mice, and this was enhanced after exercise (Figure 3E, 3F). These results suggested that the hepatic antioxidant capacities of the AD mice were reduced and that exercise enhanced the antioxidant capacities of the livers of the AD mice.

### Treadmill exercise awakened the hepatic Kupffer cells in the AD mice and enhanced their ability to hydrolyze LPS

**Figure 4:**
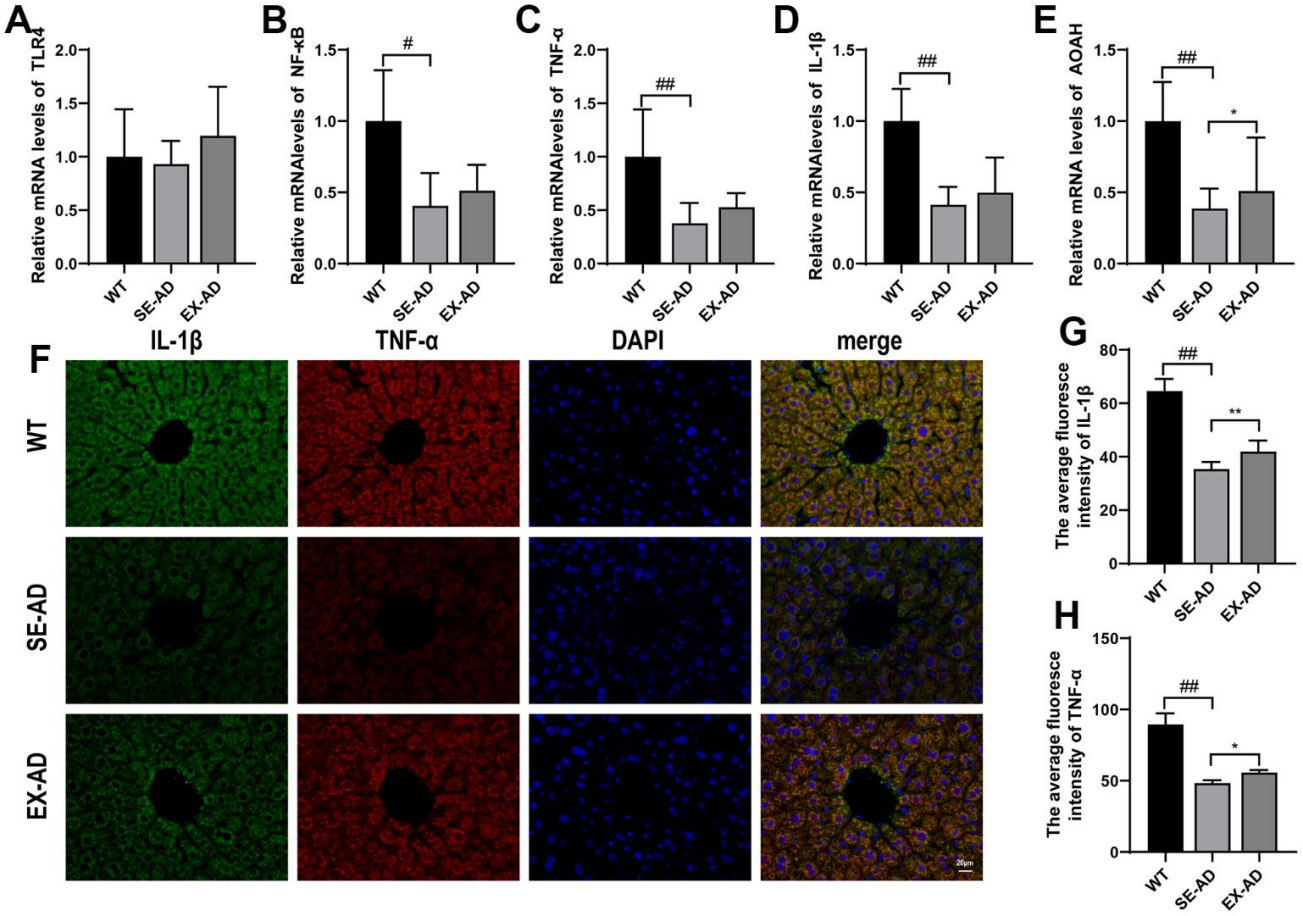
**Inflammation-related factors and AOAH expression in the animals’ livers.** (**A**) TLR4 mRNA relative expressions. (**B**) NF-κB mRNA relative expressions. (**C**) TNF-α mRNA relative expressions. (**D**) IL-1β mRNA relative expressions. (**E**) AOAH mRNA relative expressions (n = 6 in each group). (**F**) Representative images of IL-1β and TNF-α immunofluorescence staining in the animals’ livers. (**G**) Quantitative statistics of the mean fluorescence intensity of IL-1β. (**H**) Quantitative statistics of the mean fluorescence intensity of TNF-α (n = 4 in each group). The data are expressed as means ± SDs. # p < 0.05 and ## p < 0.01 compared with the WT group, and * p < 0.05 and ** p < 0.01 compared with the SE-AD group.

Hepatic Kupffer cells undergo a respiratory burst upon exposure to pathogenic microorganisms or LPS from the gut, and this may also trigger immune inflammation [23]. Compared to the wild-type mice, the TLR4/NF-κB inflammatory pathways in the livers of the AD mice were in suppressed states (Figure 4A, 4B), and the downstream inflammatory factor IL-1β and the TNF-α gene were in low expression states (Figure 4C, 4D). The LPS that reached the liver was detoxified by the AOAH in the Kupffer cells [24], and hepatic AOAH expression was found to be significantly lower in the AD mice than in the wild-type mice. These results suggested that the hepatic Kupffer cells in the AD mice were in a suppressed state and their response to LPS was diminished. In contrast, exercise increased TLR4/NF-κB pathway gene expression and, to some extent, IL-1β and TNF-α protein contents (Figure 4G, 4H) and AOAH expression (Figure 4E). These results suggested that exercise awakened the hepatic Kupffer cells in the AD mice and enhanced their ability to hydrolyze LPS.

### Treadmill exercise activated the hepatic Nrf2 antioxidant system in the AD mice

**Figure 5:**
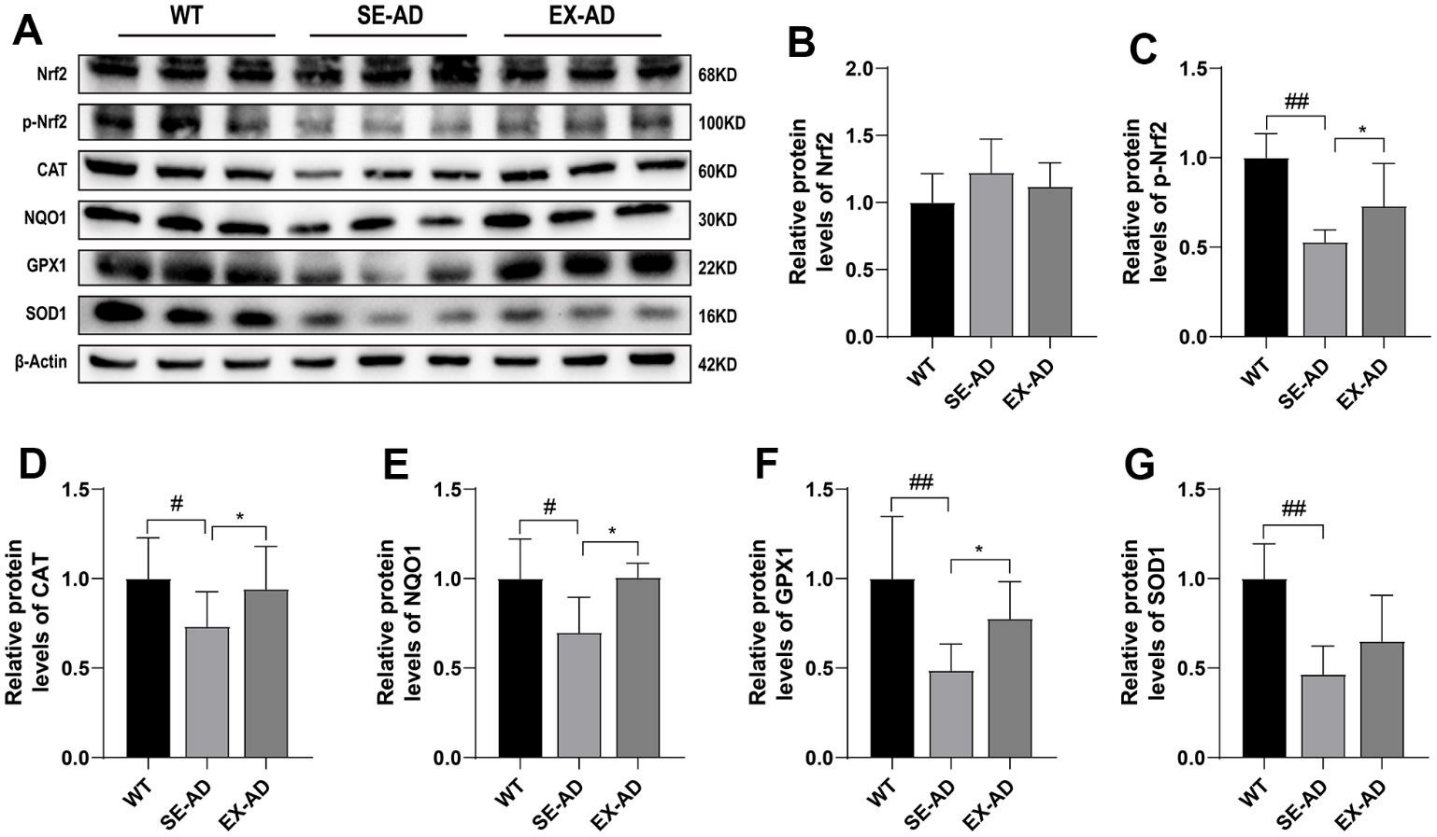
**Nrf2 and antioxidant protein expression in the mouse livers.** (**A**) Representative images of the protein immunoblots. (**B**) Relative contents of Nrf2 protein. (**C**) Relative contents of phosphorylated Nrf2 protein. (**D**) Relative contents of CAT protein. (**E**) Relative contents of NQO1 protein. (**F**) Relative contents of GPX1 protein. (**G**) Relative contents of SOD1 protein (n = 5 for each group). The data are presented as means ± SDs. # p < 0.05 and ## p < 0.01 compared with the WT group, and * p < 0.05 compared with the SE-AD group.

Nuclear factor erythroid 2-related factor 2 (Nrf2), a reactive oxygen species-sensitive transcription factor, is a master regulator of antioxidant defense and regulates more than 200 cytoprotective genes in response to oxidative stress [25]. As shown in Figure 5, Nrf2 depletion was not present in the livers of the AD mice and was instead higher compared to the wild-type mice (Figure 5B). However, the phosphorylation of Nrf2 was significantly lower in the AD mice compared to the wild-type mouse, and exercise promoted the phosphorylation of Nrf2 in the AD mice (Figure 5C). The expressions of the antioxidant proteins CAT, NQO1, GPX1, and NQO1, which are located downstream of Nrf2, were significantly lower in the livers of the AD mice than in the wild-type mice, and this was enhanced after exercise. These results suggested that exercise activated the antioxidant system of hepatic Nrf2 in the AD mice.

### Exercise protected the gut barrier and reduced LPS displacement in the AD mice

**Figure 6:**
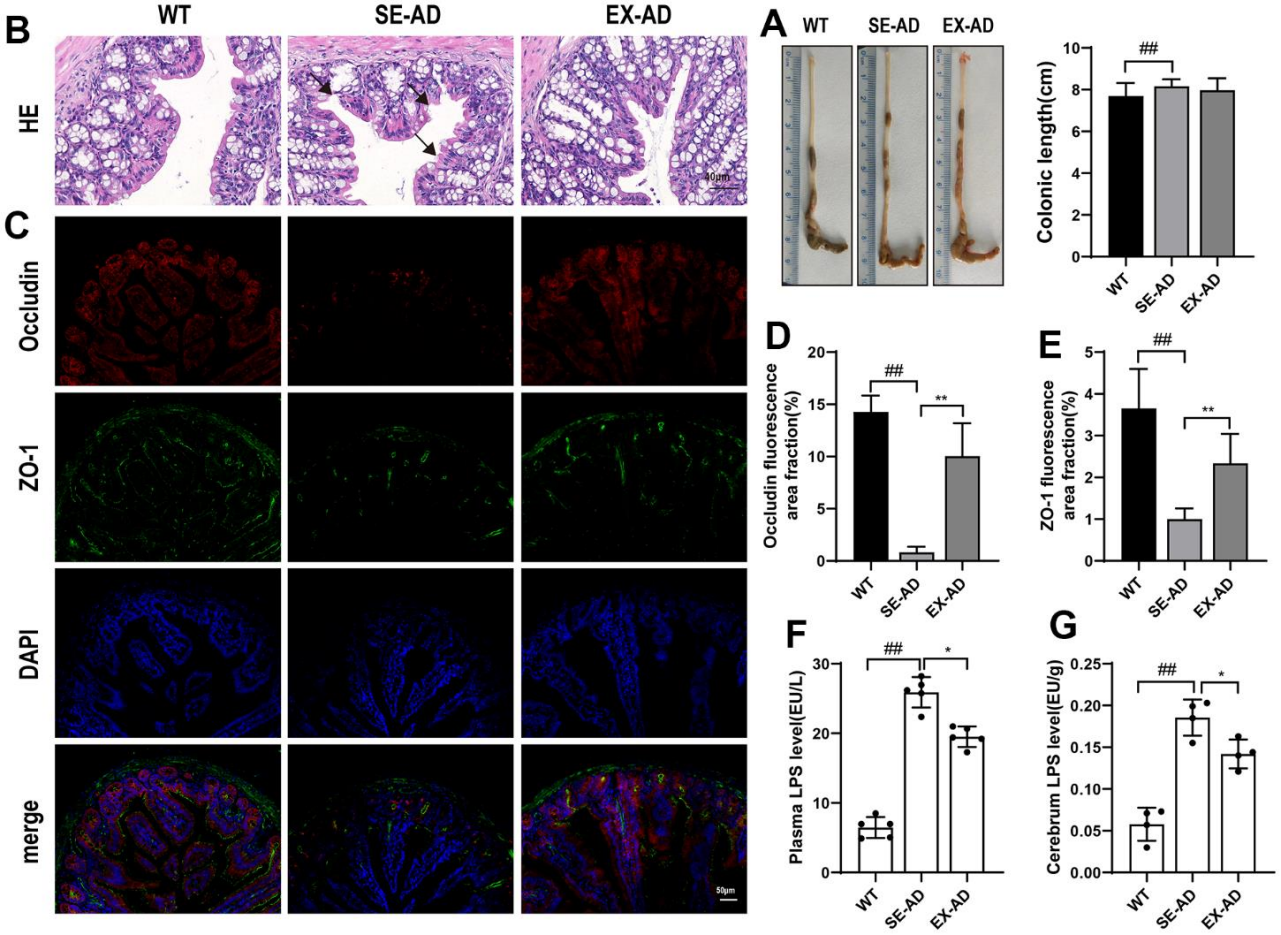
**Mouse intestinal barrier and LPS displacement.** (**A**) Representative images and length statistics of the animals’ colons (n = 12 per group). (**B**) Representative images of the colon HE staining (n = 4 per group). (**C**) Representative images of the intestinal ZO-1 and occludin immunofluorescence (n = 4 per group). (**D**) Quantitative statistics of the occludin fluorescence area. (**E**) Quantitative statistics of the ZO-1 fluorescence area. (**F**) LPS contents in serum samples (n = 6 per group). (**G**) LPS contents in the animals’ brains (n = 4 per group). The data are expressed as means ± SDs. # p < 0.05 and ## p < 0.01 compared with the WT group, and * p < 0.05 and ** p < 0.01 compared with the SE-AD group.

Molecules such as nutrients and pathogens from the intestine enter the liver through the portal vein, which supplies the main blood to the liver [26]. We observed the gut barrier and the LPS displacement in the AD mice to investigate the possible sources of liver injury. The colonic lengths of the AD mice were longer than those of the wild-type mice, and exercise had no significant effects on it (Figure 6A). The HE staining showed that the colonic mucosae of the wild-type mice were smooth and intact while the AD mice had broken colonic mucosae, as shown by the black arrow in the figure. The colonic mucosae were relatively intact but still thin after the exercise intervention (Figure 6B). We had previously found a significant decrease in the colonic barrier proteins ZO-1 and occludin in AD mice [20], and here, we observed changes in the intestinal barrier proteins ZO-1 and occludin and found that the intestinal barrier proteins ZO-1 and occludin were similarly significantly decreased in the AD mice compared to the wild type mice, and they were significantly increased after exercise (Figure 6D, 6E). The LPS levels were significantly increased in the blood samples and brains of the AD mice compared to the wild-type mice while they were significantly decreased in the blood samples and brains after exercise. These results suggested that exercise protected the intestinal barrier and reduced LPS displacement in the AD mice.

## DISCUSSION

Appropriate physical exercise can have beneficial effects on the brain and improve learning and memory abilities in AD patients, and this has been widely studied and confirmed, but the involvement of peripheral organs in the beneficial regulation of the brain during this process has rarely been studied. We observed the effects of treadmill exercise on liver injuries and LPS accumulation in the brains of APP/PS1 transgenic AD mice, and we investigated the potential mechanism of action of the gut– liver–brain axis. The main findings were as follows: (1) There were liver function abnormalities in the AD mice, and treadmill exercise alleviated the liver damage in the AD mice. (2) Treadmill exercise alleviated the hepatic oxidative stress in the AD mice. (3) Kupffer cells were involved in the hepatic oxidative stress in both the wild-type and AD mice. (4) The Kupffer cells in the AD mice were in a suppressed state, with significantly lower prooxidant, immune inflammation, and ability to hydrolyze LPS than those in the wild-type mice, and treadmill exercise reversed this situation. (5) Treadmill exercise promoted hepatic Nrf2 phosphorylation in the AD mice, activated the Nrf2 antioxidant and GSH systems, and improved hepatic antioxidant capacity. (6) Treadmill exercise protected the intestinal barriers of the AD mice and reduced the accumulation of LPS in their blood and brains. These results supported that treadmill exercise modulates hepatic redox homeostasis in AD to reduce oxidative damage and reduces LPS leakage by protecting the intestinal barrier while enhancing hepatic Kupffer cells to metabolize LPS to reduce its accumulation in the brain.

The liver is the main organ responsible for systemic metabolic control and metabolic detoxification and has an important role in Aβ clearance [27, 28]. Liver dysfunction may increase the risk of AD [29]. Numerous clinical studies have shown that alterations in liver metabolism or dysfunction may precede progressive cognitive decline and other typical symptoms of AD [30]. Zheng et al. used tissue-specific metabolomic analysis and found that the liver is the first organ to show metabolic dysfunction during the progression of AD pathogenesis [31]. A clinical study by Lu et al. showed that abnormalities in the liver function-related enzymes AST and ALT were associated with an increased risk of AD [32]. Liver tissues from AD patients also contain less Aβ than healthy individuals, indicating an impaired ability of the liver to take up Aβ [33]. These findings have provided key evidence that liver dysfunction is an early pathological event in AD. AST levels in blood serum are low under normal conditions, but when hepatocytes are damaged, cell membrane permeability increases and intracytoplasmic AST is released into the blood, leading to an increase in the concentration of AST in blood serum [34]. In this study, we found that AST and ALT levels were significantly elevated in the sera of the AD mice, and in addition, the liver pathology showed the presence of a large number of Kupffer cell aggregates and sticky and indistinct boundaries between liver cells, indicating that the AD mice had abnormal liver function, as is seen in AD patients. The AST and ALT levels in the sera of the mice in the AD-EX group decreased and their liver pathology was improved, suggesting that treadmill exercise improved the liver dysfunction of the AD mice.

Since we found an abnormal aggregation of Kupffer cells in the liver pathology of the AD mice, it was hypothesized that the liver injuries in the AD mice may have been related to the Kupffer cells, which are macrophages in the liver and are the largest population of innate immune cells in the liver. In the liver, Kupffer cells account for approximately 20–25% of non-parenchymal cells [35]. The high occupancy means that hepatic Kupffer cells play a crucial role in maintaining liver function and homeostasis in the body. The multiple ways that Kupffer cells recognize receptors help to identify and eliminate invading foreign pathogens [36]. Excess reactive oxygen species and immune cytokines may be produced during this process, leading to oxidative stress and inflammation in the liver. In this study, highly consistent changes in the fluorescence intensities of CD68 and p47 phox immunofluorescence co-localization were found, confirming that Kupffer cells play an important role in hepatic oxidative stress. The protein carbonyl and MDA contents were significantly elevated in the livers of the AD mice, indicating that proteins and lipids were over-oxidized and the liver was under oxidative stress. However, the prooxidant genes were tested and were not found to be elevated, but rather, they were decreased compared to the wild-type mice. Further detection of the TLR4/NF-κB pathway and the downstream expression of the inflammatory factors IL-1β and TNF-α were significantly lower in the AD mice than in the wild-type mice, thus indicating that the hepatic Kupffer cells in the AD mice were in a state of oxidative stress and suppressed immune activation. This was similar to the study by Zheng et al., where they found that the livers of 5-month-old APP/PS1 mice were in hypometabolic states [31]. Treadmill exercise increased the expression of prooxidant genes and inflammatory pathway genes in the livers of the AD mice, to some extent, suggesting that treadmill exercise activated or awakened the Kupffer cells. The expression of AOAH, a hydrolase of LPS in Kupffer cells, was further examined, and as hypothesized, the hepatic AOAH levels in the AD mice were significantly lower than normal levels, and they were elevated after exercise. The above indicated that treadmill exercise alleviated hepatic oxidative damage in the AD mice, increased the activity of hepatic Kupffer cells, and enhanced their ability to metabolize LPS. However, whether this effect of exercise on hepatic Kupffer cells resulted from the attenuation of oxidative stress by exercise or if other beneficial effects of exercise on the liver caused such changes in the Kupffer cells is worth further investigation.

Under normal conditions, a balance between prooxidants and antioxidants can make an organism free from oxidative stress. We examined the hepatic antioxidant systems in AD mice and found that the hepatic GSH contents were significantly decreased and the total antioxidant capacities were reduced in the AD mice. Notably, the genes involved in GSH production, Gss, Gsr, Gclc, and Gclm, did not change significantly, and the decrease in GSH contents did not come from insufficient production but may have been related to increased consumption, which deserves further study to confirm at a later stage. Recent studies have revealed that Nrf2, a transcription factor, is considered a key regulator of cellular resistance to oxidative stress [37, 38]. We explored this and found that Nrf2 levels in the livers of the AD mice were not depleted and instead had a tendency to increase, but the phosphorylation of Nrf2 was reduced and the expressions of the downstream antioxidant proteins CAT, NQO1, GPX1, and NQO1 were significantly reduced, indicating that the Nrf2 antioxidant systems in the livers of the AD mice were also in suppressed states. This indicated that the hepatic Nrf2 antioxidant systems of the AD mice were also in suppressed states, and treadmill exercise promoted Nrf2 phosphorylation and downstream antioxidant protein expression in the livers of the AD mice. Appropriate exercise could improve antioxidant capacity, and this was consistent with numerous recent studies [39–41]. Prooxidant and antioxidant levels in the livers of the AD mice were lower than those in normal mice, but there was oxidative damage, and treadmill exercise increased both the prooxidant and antioxidant levels but reduced oxidative stress, suggesting that treadmill exercise may have regulated the hepatic redox balances in the AD mice, ultimately reducing oxidative stress.

There is a natural anatomical link between the intestine and the liver, and there is substantial epidemiological evidence for a close association between the intestine and the liver [23]. Our observation of colonic pathomorphology revealed broken colonic barriers in the AD mice in addition to significant downregulations of the ZO-1 and occludin proteins in the intestines. Intestinal barrier function in the AD mice was low, which was consistent with numerous recent studies [42, 43]. The tight junctions of ZO-1 and occludin form a barrier that serves as a valve for the entry of macromolecules such as LPS into the blood, and its downregulation can lead to the excessive translocation of intestinal LPS into the blood. We also observed a significant elevation of LPS levels in the sera of the AD mice, whereas after performing treadmill exercise, their intestinal barrier function was improved and the LPS levels in the sera were significantly reduced. After the blood carrying LPS was injected into the liver through the portal vein, it was phagocytosed and detoxified by the Kupffer cells, but the hepatic Kupffer cells of the AD mice were in an inhibited state and the hydrolase AOAH of the LPS was significantly decreased, and thus, this led to more LPS leaving the liver and circulating in the animals’ bodies, which eventually led to severe accumulations of LPS in the brains of the AD mice. Clinical studies have found higher levels of LPS in the brains of AD patients compared to normal brains, localized in Aβ plaques and around cerebral blood vessels [44]. Long-term infusions of LPS into the brains of mice have shown inflammatory and pathological changes similar to those of AD patients [45], and multiple intraperitoneal injections of LPS into mice have resulted in increased Aβ levels in the hippocampus and exacerbated cognitive deficits [46]. Therefore, LPS is closely associated with the deterioration seen in AD, and treadmill exercise reduces the accumulation of LPS in the brain by modulating the intestinal barrier and hepatic Kupffer cells, which is important for slowing down the further deterioration of brain pathology that is seen in AD patients. However, how much of the benefit of exercise on the brains of AD patients comes from the reductions in LPS remains to be further confirmed. In addition, LPS is a major component of the cell walls of Gram-negative bacteria in intestinal microbes, and maintaining the ecological balance of intestinal microbes can reduce the production of LPS [47, 48]. Our previous study found that treadmill exercise enriched intestinal microbes, reduced harmful bacteria, and protected the blood–brain barrier in AD mice [20], suggesting that treadmill exercise may regulate various aspects of the microbial–gut–liver–brain axis to reduce the entry of LPS into the brain, but the magnitude of the effect of exercise on these aspects and the causal relationship between these aspects remain to be confirmed by further studies.

Interestingly, Cheng et al. [49] found that approximately 13.9% of Aβ42 and 8.9% of Aβ40 were eliminated upon flow through the liver, and this clearance was reduced with the downregulation of the receptor protein for Aβ and recombinant low-density lipoprotein receptor-related protein 1 (LRP1) in the hepatocytes of aged animals. Knocking out the LRP-1 gene reduced the clearing efficiency of Aβ by the liver and exacerbated the Aβ burden and cognitive deficits of the brain while LRP-1 overexpression enhanced the clearing efficiency of Aβ by the liver and attenuated the Aβ deposition in the brain and the cognitive deficits of APP/PS1 mice. This suggests that liver health has a profound effect on the brains of AD patients, but whether treadmill exercise improves brain pathology by promoting the hepatic clearance of Aβ is a question worth further investigation. Our findings indicated that treadmill exercise ameliorated liver injuries in AD mice, and although there may have been mechanisms of injury other than the oxidative stress, this provides a basis for studying the livers of AD patients. In addition, the causal relationship and interplay between the gut, liver, and brain in AD mice is also a question worth continuing to explore in the future, and it will likely explain why the livers of AD patients are in hypometabolic states and whether these inhibited states of Kupffer cells are related to excess LPS from the intestines. Treatments from these pathways will likely provide new prevention and rehabilitation options with therapeutic effects for AD patients.

**Figure 7:**
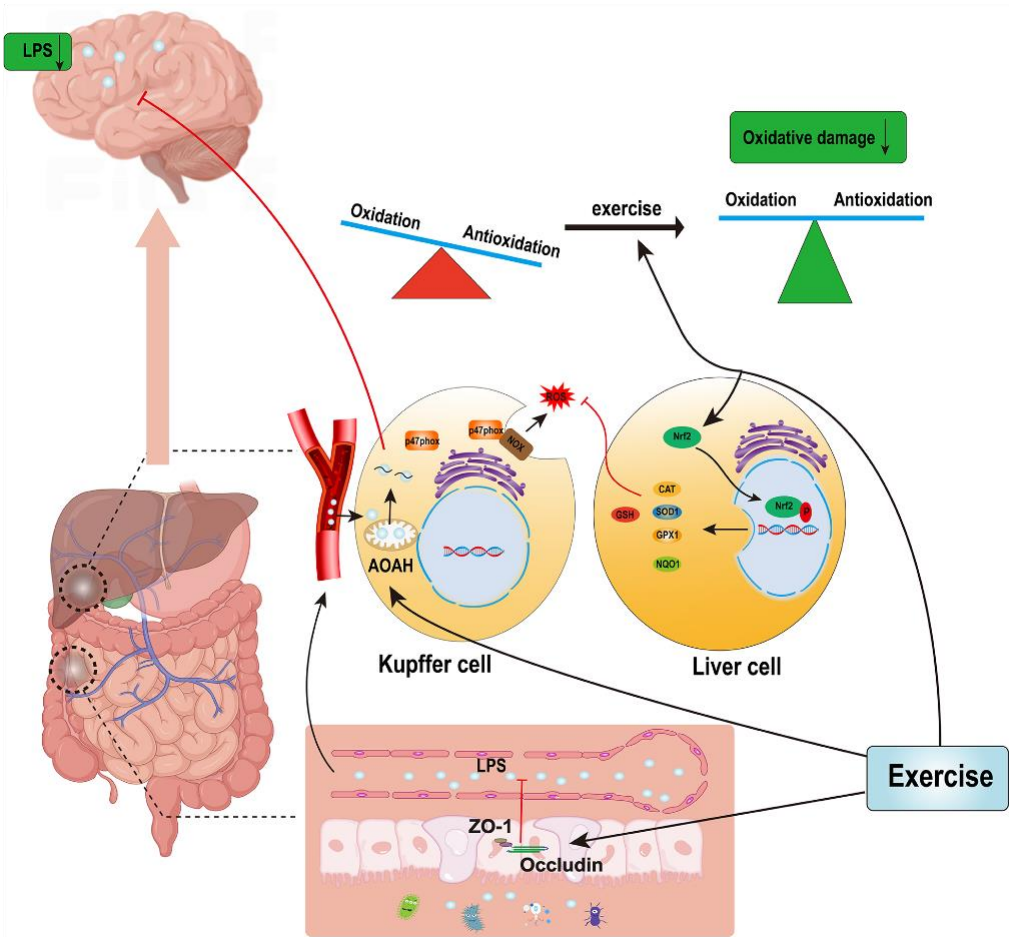
Possible mechanisms by which exercise reduces hepatic oxidative stress and LPS accumulations in the brains of AD patients.

In conclusion, the livers of AD mice were under oxidative stress, but their prooxidant and antioxidant levels were lower than those of wild-type mice, and long-term treadmill exercise reduced the oxidative stress in the livers of the AD mice and increased their prooxidant and antioxidant levels. This may have improved the oxidative damage by reshaping the redox balance, and treadmill exercise reduced the accumulation of LPS in the brains of the AD mice by simultaneously regulating each aspect of the gut–liver–Kupffer cells. There may be a potential mechanism for the beneficial effects of exercise on the brains of AD patients, as shown below in Figure 7, and it is hypothesized that LPS may be an important peripheral target molecule for exercise for improving the health of AD patients.

## MATERIALS AND METHODS

### Experimental animals and their grouping

We randomly divided 24 SPF level 3-month-old male APP/PS1 transgenic AD mice into sedentary (SE-AD) and exercise (EX-AD) groups, and 12 C57BL/6 mice were used as the wild-type (WT) control. These animals were purchased from Changzhou Cavins Laboratory Animal Co., Ltd. (Changzhou, China, license number SCXK (Su) 2016-0010) and kept in a constant temperature animal room at 22~25° C, with free access to food and water and 12 h light and dark alternating conditions.

### Experiment design

**Figure 8:**
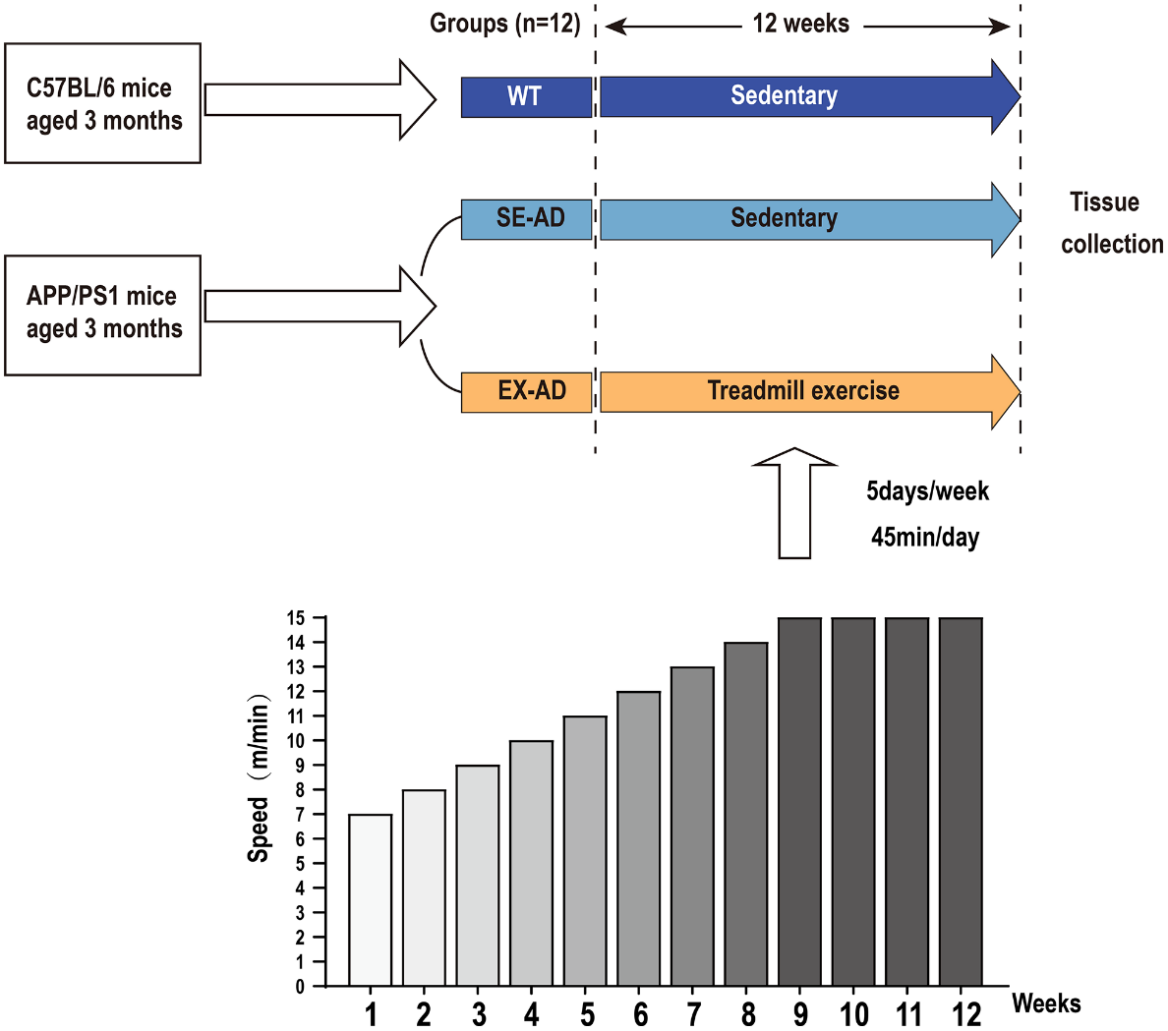
**Experiment design.** The APP/PS1 mice were divided into a serene group and an exercise group, and the C57BL/6 mice were used as the control. The exercise group was exercised by treadmill for 12 weeks, with the speed increasing from 7 m/min to 14 m/min from weeks 1 to 8 and remaining constant at 15 m/min from 9 to 12 weeks. Exercise was performed 5 days per week for 45 min each time.

The mice in the SE-AD group were exercised on a treadmill for 12 weeks, and the exercise scheme was as we reported previously, with the speed increasing from 7 m/min to 14 m/min from weeks 1 to 8 and remaining constant at 15 m/min from weeks 9 to 12 (as shown in Figure 8), with 5 days of exercise per week and rest on Thursdays and Sundays, with daily exercise fixed at the time period of 17:00–18:00 for 45 min each time. The WT group was placed on a stationary treadmill with the SE-AD group during the training time of the exercise group [20]. Our preliminary results showed that the exercise protocol was effective at reducing pathology of the brain in the AD mice [50].

### Tissue collection

After the last exercise, 12 h of fasting was performed, during which free access to water was allowed. Blood was taken from the animals’ hearts with a syringe containing sodium heparin after anesthesia with isoflurane and centrifuged, and the supernatant was aspirated and stored at −80° C. We took tissues from the mouse brains and livers in a lyophilization tube, put them into liquid nitrogen quickly, and then stored them in a −80° C refrigerator. The livers, colons, and intestinal tissues of 4 mice in each group were randomly selected and fixed in 4% paraformaldehyde solution for 48 h, followed by slice preparation for HE staining and immunofluorescence detection.

### HE staining

The fixed liver and colon tissues were routinely dehydrated, transparent, wax-impregnated, and paraffin-embedded to make 5 μm slices, and then the slices were dewaxed and hydrated, stained with hematoxylin-eosin, dehydrated and transparently sealed, and, finally, placed under a microscope for observation and image acquisition.

### Biochemical test

The serum after centrifugation was taken, and ALT and AST kits (Quanzhou Ruixin Biotechnology Co., Ltd., item nos. RXWB0374 and RXSH0649, respectively, Quanzhou, China) were used to test the serum ALT and AST contents by strictly following the steps in the instruction manuals. The mouse liver tissues were ground and the supernatant was aspirated, and a protein carbonyl content kit, glutathione (GSH) content kit, malondialdehyde (MDA) kit, and total antioxidant capacity (T-AOC) kit (Quanzhou Ruixin Biotechnology Co., Ltd., item nos. RXSH0589, RXWB0005, RXWB0113, and RXWB0296, respectively) were used to detect the liver protein carbonyl, MDA, and GSH contents and the total antioxidant capacity by strictly following the steps in the instruction manuals.

### ELISA assay

The animals’ serum samples and brain tissues were analyzed by an LPS ELISA kit (Quanzhou Ruixin Biotechnology Co., Ltd., RX202425M), which was operated according to the instruction procedures. The absorbance (OD) values were measured at 450 nm using an enzyme standardization instrument, and finally, a standard curve was established to calculate the LPS contents in the serum samples and brain tissues of each group of mice.

### Immunofluorescence assays

Wax blocks were cut using a cutter into slices with thicknesses of 5 μm, and 3 slices were selected for each mouse, with each slice positioned 100 μm apart. The paraffin slices were dewaxed and rehydrated for antigen repair. The slides were placed in PBS and washed 3 times to remove the excess solution from the surface of the slices. They were then placed in an autofluorescence quencher for 5 min, rinsed under running water, and incubated with BSA dropwise for 20 min. This was followed by primary antibody incubation, and the primary antibodies were CD68 (Servicebio, GB113109, 1:100, Wuhan, China), p47 phox (Servicebio, GB11724, 1:300, Wuhan, China), TNF-α (Proteintech, 60291-1-lg, 1:100, Wuhan, China), IL-1β (ABclonal, A1112, 1:100, Wuhan, China), ZO-1 (Servicebio, GB111402, 1:500, Wuhan, China), and occludin (Servicebio, GB111401, 1:1000, Wuhan, China). These primary antibodies were incubated overnight and secondary antibodies were performed the next day, protected from light (Servicebio, GB21301, GB25303, 1:300, 1:400), and incubated for 1 h. The excess secondary antibodies were washed away and dried at a low temperature in a desiccator, and then the slices were sealed with a sealer containing DAPI (SouthernBiotech, 0100-20, Birmingham, AL, USA). Images were observed and acquired under a microscope, and the fluorescence area and average fluorescence intensity were analyzed by Image J software (version 5.0).

### Real-time PCR assay

The total RNA was extracted from the mouse brain tissues (approximately 30 mg) according to Thermo Fisher Scientific’s Trizol reagent instructions (Thermo Fisher Scientific, 15596018, Waltham, MA, USA). A MiniAmp PCR instrument was used to perform the reverse transcription according to the instructions of the reverse transcription kit (Servicebio, G3337, Wuhan, China). We configured 20 μL of the expansion system according to the instructions of the Servicebio qPCR kit (Servicebio, G3320, Wuhan, China), and the expansion was completed using a BIO-RAD Real-Time PCR instrument. Finally, based on the detected Ct values, the relative expression of the target gene mRNA was calculated using the 2−∆∆CT method with GAPDH as the internal reference gene, and the data were normalized for graphing after statistical analysis. The primer sequences of the target genes used are listed in Table 1.

**Table 1:** Primer sequences of target genes.

**Gene**	**Forward primer**	**Reverse primer**
TLR4	ACAAACGCCGGAACTTTTCG	GTCGGACACACACAACTTAAGC
NF-κB	ATCATCGAACAGCCGAAGCA	TGATGGTGGGGTGTGTCTTG
TNF-α	TTAGAAAGGGGATTATGGCTCA	ACTCTCCCTTTGCAGAACTCAG
p47phox	ACACCTTCATTCGCCATATTGC	TCGGTGAATTTTCTGTAGACCAC
Nox4	TCCATCAAGCCAAGATTCTGAG	GGTTTCCAGTCATCCAGTAGAG
IL-1β	CTCACAAGCAGAGCACAAGC	AGCTGTCTGCTCATTCACGA
Gss	CAAAGCAGGCCATAGACAGGG	AAAAGCGTGAATGGGGCATAC
Gsr	CACGGCTATGCAACATTCGC	GTGTGGAGCGGTAAACTTTTTC
Gclc	GGGGTGACGAGGTGGAGTA	GTTGGGGTTTGTCCTCTCCC
Gclm	AGGAGCTTCGGGACTGTATCC	GGGACATGGTGCATTCCAAAA
AOAH	ATGAAGGCTGATGTGGTGTG	AGGACCTCCTGAGGACTTGT
GAPDH	CATGGCCTTCCGTGTTCCTA	CCTGCTTCACCACCTTCTTGAT

### Western blotting assay

We added 50 mg of liver tissue from each group of mice to 500 μL of RIPA lysate (Servicebio, G2020, Wuhan, China), 10 μL of cocktail (Servicebio, G2006, Wuhan, China), and 5 μL of phosphorylated protease inhibitor (Servicebio, G2007, Wuhan, China) for homogenization. The solution was then centrifuged to obtain the supernatant, and subsequently, we quantified the protein using the BCA protein concentration assay kit (Beyotime, P00125, Shanghai, China) according to the instructions. Precast gel (Abiowell, AWB1010, Changsha, China) was used for the electrophoresis, followed by membrane transfer, sealing, and incubation overnight at 4° C with the primary antibodies Nrf2 (Proteintech, 16396-1-AP, 1:5000, Wuhan, China), phosphorylated Nrf2 (ABclonal, AP1133, 1:1000, Wuhan, China), CAT (Proteintech, 21260-1-AP, 1:5000, Wuhan, China), NQO1 (Proteintech, 11451-1-AP, 1:2000, Wuhan, China), GPX1 (Proteintech, 29329-1-AP, 1:2000, Wuhan, China), SOD1 (Proteintech, 10269-1-AP, 1:2000, Wuhan, China), and β-actin (ABclonal, AC026, 1:50000, Wuhan, China). The next day, the samples were washed 3 times with PBST for 10 min each time, and the secondary antibody (Proteintech, 20000374, 1:5000, Wuhan, China) was incubated at room temperature for 1 h. The samples were washed 3 times with PBST for 10 min each time, and the ECL luminescence kit (Servicebio, G2014, Wuhan, China) was used to produce photographs on a Tanon-5200 gel system. Image J software (version 5.0). was used to analyze the integrated grayscale values and count the relative protein expressions.

### Statistical analysis

All data were expressed as mean ± standard deviation (Mean ± SD), and were analyzed and plotted using Graphed Prism 8.0 software. The statistical method was one-way ANOVA, and the least significant difference (LSD) method was used for post hoc multiple comparisons. p<0.05 was the significant difference, and p<0.01 was the extremely significant difference.

## References

[1] New insights into the genetic etiology of Alzheimer’s disease and related dementias. Nat Genet. 2022; 54:412–36. 10.1038/s41588-022-01024-z 35379992 PMC9005347

[2] Eratne D, Loi SM, Farrand S, Kelso W, Velakoulis D, Looi JC. Alzheimer’s disease: clinical update on epidemiology, pathophysiology and diagnosis. Australas Psychiatry. 2018; 26:347–57. 10.1177/1039856218762308 29614878

[3] Hu X, Wang T, Jin F. Alzheimer’s disease and gut microbiota. Sci China Life Sci. 2016; 59:1006–23. 10.1007/s11427-016-5083-9 27566465

[4] Ren R, Qi J, Lin S, Liu X, Yin P, Wang Z, Tang R, Wang J, Huang Q, Li J, Xie X, Hu Y, Cui S, et al. The China Alzheimer Report 2022. Gen Psychiatr. 2022; 35:e100751. 10.1136/gpsych-2022-100751 35372787 PMC8919463

[5] Prince M, Bryce R, Albanese E, Wimo A, Ribeiro W, Ferri CP. The global prevalence of dementia: a systematic review and metaanalysis. Alzheimers Dement. 2013; 9:63–75.e2. 10.1016/j.jalz.2012.11.007 23305823

[6] Feng X, Provenzano FA, Small SA, and Alzheimer’s Disease Neuroimaging Initiative. A deep learning MRI approach outperforms other biomarkers of prodromal Alzheimer’s disease. Alzheimers Res Ther. 2022; 14:45. 10.1186/s13195-022-00985-x 35351193 PMC8966329

[7] Liu Q, Xie T, Xi Y, Li L, Mo F, Liu X, Liu Z, Gao JM, Yuan T. Sesamol Attenuates Amyloid Peptide Accumulation and Cognitive Deficits in APP/PS1 Mice: The Mediating Role of the Gut-Brain Axis. J Agric Food Chem. 2021; 69:12717–29. 10.1021/acs.jafc.1c04687 34669408

[8] Nho K, Kueider-Paisley A, Ahmad S, MahmoudianDehkordi S, Arnold M, Risacher SL, Louie G, Blach C, Baillie R, Han X, Kastenmüller G, Trojanowski JQ, Shaw LM, et al, and Alzheimer’s Disease Neuroimaging Initiative and the Alzheimer Disease Metabolomics Consortium. Association of Altered Liver Enzymes With Alzheimer Disease Diagnosis, Cognition, Neuroimaging Measures, and Cerebrospinal Fluid Biomarkers. JAMA Netw Open. 2019; 2:e197978. 10.1001/jamanetworkopen.2019.7978 31365104 PMC6669786

[9] Khan MS, Ikram M, Park JS, Park TJ, Kim MO. Gut Microbiota, Its Role in Induction of Alzheimer’s Disease Pathology, and Possible Therapeutic Interventions: Special Focus on Anthocyanins. Cells. 2020; 9:853. 10.3390/cells9040853 32244729 PMC7226756

[10] Liu L, Wang H, Chen X, Xie P. Gut microbiota: a new insight into neurological diseases. Chin Med J (Engl). 2023; 136:1261–77. 10.1097/CM9.0000000000002212 35830286 PMC10309523

[11] Ling Z, Zhu M, Yan X, Cheng Y, Shao L, Liu X, Jiang R, Wu S. Structural and Functional Dysbiosis of Fecal Microbiota in Chinese Patients With Alzheimer’s Disease. Front Cell Dev Biol. 2021; 8:634069. 10.3389/fcell.2020.634069 33614635 PMC7889981

[12] Zhao Y, Jaber V, Lukiw WJ. Secretory Products of the Human GI Tract Microbiome and Their Potential Impact on Alzheimer’s Disease (AD): Detection of Lipopolysaccharide (LPS) in AD Hippocampus. Front Cell Infect Microbiol. 2017; 7:318. 10.3389/fcimb.2017.00318 28744452 PMC5504724

[13] Tuin A, Huizinga-Van der Vlag A, van Loenen-Weemaes AM, Meijer DK, Poelstra K. On the role and fate of LPS-dephosphorylating activity in the rat liver. Am J Physiol Gastrointest Liver Physiol. 2006; 290:G377–85. 10.1152/ajpgi.00147.2005 16223948

[14] Lukiw WJ. Gastrointestinal (GI) Tract Microbiome-Derived Neurotoxins-Potent Neuro-Inflammatory Signals From the GI Tract via the Systemic Circulation Into the Brain. Front Cell Infect Microbiol. 2020; 10:22. 10.3389/fcimb.2020.00022 32117799 PMC7028696

[15] Pan D, Li G, Jiang C, Hu J, Hu X. Regulatory mechanisms of macrophage polarization in adipose tissue. Front Immunol. 2023; 14:1149366. 10.3389/fimmu.2023.1149366 37283763 PMC10240406

[16] Mantovani A, Biswas SK, Galdiero MR, Sica A, Locati M. Macrophage plasticity and polarization in tissue repair and remodelling. J Pathol. 2013; 229:176–85. 10.1002/path.4133 23096265

[17] Huang Z, Lin HWK, Zhang Q, Zong X. Targeting Alzheimer’s Disease: The Critical Crosstalk between the Liver and Brain. Nutrients. 2022; 14:4298. 10.3390/nu14204298 36296980 PMC9609624

[18] Aczel D, Gyorgy B, Bakonyi P, BukhAri R, Pinho R, Boldogh I, Yaodong G, Radak Z. The Systemic Effects of Exercise on the Systemic Effects of Alzheimer’s Disease. Antioxidants (Basel). 2022; 11:1028. 10.3390/antiox11051028 35624892 PMC9137920

[19] Key MN, Szabo-Reed AN. Impact of Diet and Exercise Interventions on Cognition and Brain Health in Older Adults: A Narrative Review. Nutrients. 2023; 15:2495. 10.3390/nu15112495 37299458 PMC10255782

[20] Yuan S, Yang J, Jian Y, Lei Y, Yao S, Hu Z, Liu X, Tang C, Liu W. Treadmill Exercise Modulates Intestinal Microbes and Suppresses LPS Displacement to Alleviate Neuroinflammation in the Brains of APP/PS1 Mice. Nutrients. 2022; 14:4134. 10.3390/nu14194134 36235786 PMC9572649

[21] Yu Y, Zhou S, Wang Y, Di S, Wang Y, Huang X, Chen Y. Leonurine alleviates acetaminophen-induced acute liver injury by regulating the PI3K/AKT signaling pathway in mice. Int Immunopharmacol. 2023; 120:110375. 10.1016/j.intimp.2023.110375 37267857

[22] Pop OT, Geng A, Flint E, Singanayagam A, Ercan C, Possamai L, Patel VC, Kuenzler P, Meier MA, Soysal S, Hruz P, Kollmar O, Tatham KC, et al. AXL Expression on Homeostatic Resident Liver Macrophages Is Reduced in Cirrhosis Following GAS6 Production by Hepatic Stellate Cells. Cell Mol Gastroenterol Hepatol. 2023; 16:17–37. 10.1016/j.jcmgh.2023.03.007 37004869 PMC10209017

[23] Zhang X, Liu H, Hashimoto K, Yuan S, Zhang J. The gut-liver axis in sepsis: interaction mechanisms and therapeutic potential. Crit Care. 2022; 26:213. 10.1186/s13054-022-04090-1 35831877 PMC9277879

[24] Lu M, Zhang M, Takashima A, Weiss J, Apicella MA, Li XH, Yuan D, Munford RS. Lipopolysaccharide deacylation by an endogenous lipase controls innate antibody responses to Gram-negative bacteria. Nat Immunol. 2005; 6:989–94. 10.1038/ni1246 16155573

[25] Dinkova-Kostova AT, Copple IM. Advances and challenges in therapeutic targeting of NRF2. Trends Pharmacol Sci. 2023; 44:137–49. 10.1016/j.tips.2022.12.003 36628798

[26] Adams DH, Eksteen B, Curbishley SM. Immunology of the gut and liver: a love/hate relationship. Gut. 2008; 57:838–48. 10.1136/gut.2007.122168 18203807

[27] Cheng Y, Tian DY, Wang YJ. Peripheral clearance of brain-derived Aβ in Alzheimer’s disease: pathophysiology and therapeutic perspectives. Transl Neurodegener. 2020; 9:16. 10.1186/s40035-020-00195-1 32381118 PMC7204069

[28] Wang J, Gu BJ, Masters CL, Wang YJ. A systemic view of Alzheimer disease - insights from amyloid-β metabolism beyond the brain. Nat Rev Neurol. 2017; 13:703. 10.1038/nrneurol.2017.147 29027541

[29] Xiang Y, Bu XL, Liu YH, Zhu C, Shen LL, Jiao SS, Zhu XY, Giunta B, Tan J, Song WH, Zhou HD, Zhou XF, Wang YJ. Physiological amyloid-beta clearance in the periphery and its therapeutic potential for Alzheimer’s disease. Acta Neuropathol. 2015; 130:487–99. 10.1007/s00401-015-1477-1 26363791 PMC4575389

[30] Kaur H, Seeger D, Golovko S, Golovko M, Combs CK. Liver Bile Acid Changes in Mouse Models of Alzheimer’s Disease. Int J Mol Sci. 2021; 22:7451. 10.3390/ijms22147451 34299071 PMC8303891

[31] Zheng H, Cai A, Shu Q, Niu Y, Xu P, Li C, Lin L, Gao H. Tissue-Specific Metabolomics Analysis Identifies the Liver as a Major Organ of Metabolic Disorders in Amyloid Precursor Protein/Presenilin 1 Mice of Alzheimer’s Disease. J Proteome Res. 2019; 18:1218–27. 10.1021/acs.jproteome.8b00847 30592618

[32] Lu Y, Pike JR, Selvin E, Mosley T, Palta P, Sharrett AR, Thomas A, Loehr L, Sidney Barritt A, Hoogeveen RC, Heiss G. Low Liver Enzymes and Risk of Dementia: The Atherosclerosis Risk in Communities (ARIC) Study. J Alzheimers Dis. 2021; 79:1775–84. 10.3233/JAD-201241 33459646 PMC8679120

[33] Roher AE, Esh CL, Kokjohn TA, Castaño EM, Van Vickle GD, Kalback WM, Patton RL, Luehrs DC, Daugs ID, Kuo YM, Emmerling MR, Soares H, Quinn JF, et al. Amyloid beta peptides in human plasma and tissues and their significance for Alzheimer’s disease. Alzheimers Dement. 2009; 5:18–29. 10.1016/j.jalz.2008.10.004 19118806 PMC2663406

[34] Prasanna PL, Renu K, Valsala Gopalakrishnan A. New molecular and biochemical insights of doxorubicin-induced hepatotoxicity. Life Sci. 2020; 250:117599. 10.1016/j.lfs.2020.117599 32234491

[35] Lopez BG, Tsai MS, Baratta JL, Longmuir KJ, Robertson RT. Characterization of Kupffer cells in livers of developing mice. Comp Hepatol. 2011; 10:2. 10.1186/1476-5926-10-2 21749715 PMC3148529

[36] Dou L, Shi X, He X, Gao Y. Macrophage Phenotype and Function in Liver Disorder. Front Immunol. 2020; 10:3112. 10.3389/fimmu.2019.03112 32047496 PMC6997484

[37] Yagishita Y, Gatbonton-Schwager TN, McCallum ML, Kensler TW. Current Landscape of NRF2 Biomarkers in Clinical Trials. Antioxidants (Basel). 2020; 9:716. 10.3390/antiox9080716 32784785 PMC7464243

[38] Márton M, Tihanyi N, Gyulavári P, Bánhegyi G, Kapuy O. NRF2-regulated cell cycle arrest at early stage of oxidative stress response mechanism. PLoS One. 2018; 13:e0207949. 10.1371/journal.pone.0207949 30485363 PMC6261604

[39] Gomez-Cabrera MC, Domenech E, Viña J. Moderate exercise is an antioxidant: upregulation of antioxidant genes by training. Free Radic Biol Med. 2008; 44:126–31. 10.1016/j.freeradbiomed.2007.02.001 18191748

[40] Mohammadkhani R, Komaki A, Karimi SA, Behzad M, Heidarisasan S, Salehi I. Maternal high-intensity interval training as a suitable approach for offspring’s heart protection in rat: evidence from oxidative stress and mitochondrial genes. Front Physiol. 2023; 14:1117666. 10.3389/fphys.2023.1117666 37288431 PMC10242028

[41] John A, Howarth FC, Raza H. Exercise alleviates diabetic complications by inhibiting oxidative stress-mediated signaling cascade and mitochondrial metabolic stress in GK diabetic rat tissues. Front Physiol. 2022; 13:1052608. 10.3389/fphys.2022.1052608 36531176 PMC9751475

[42] Zhu G, Zhao J, Wang G, Chen W. Bifidobacterium breve HNXY26M4 Attenuates Cognitive Deficits and Neuroinflammation by Regulating the Gut-Brain Axis in APP/PS1 Mice. J Agric Food Chem. 2023; 71:4646–55. 10.1021/acs.jafc.3c00652 36888896

[43] Hao X, Ding N, Zhang Y, Yang Y, Zhao Y, Zhao J, Li Y, Li Z. Benign regulation of the gut microbiota: The possible mechanism through which the beneficial effects of manual acupuncture on cognitive ability and intestinal mucosal barrier function occur in APP/PS1 mice. Front Neurosci. 2022; 16:960026. 10.3389/fnins.2022.960026 35992924 PMC9382294

[44] Zhan X, Stamova B, Jin LW, DeCarli C, Phinney B, Sharp FR. Gram-negative bacterial molecules associate with Alzheimer disease pathology. Neurology. 2016; 87:2324–32. 10.1212/WNL.0000000000003391 27784770 PMC5135029

[45] Hauss-Wegrzyniak B, Vraniak PD, Wenk GL. LPS-induced neuroinflammatory effects do not recover with time. Neuroreport. 2000; 11:1759–63. 10.1097/00001756-200006050-00032 10852239

[46] Kahn MS, Kranjac D, Alonzo CA, Haase JH, Cedillos RO, McLinden KA, Boehm GW, Chumley MJ. Prolonged elevation in hippocampal Aβ and cognitive deficits following repeated endotoxin exposure in the mouse. Behav Brain Res. 2012; 229:176–84. 10.1016/j.bbr.2012.01.010 22249135

[47] Lin TL, Shu CC, Chen YM, Lu JJ, Wu TS, Lai WF, Tzeng CM, Lai HC, Lu CC. Like Cures Like: Pharmacological Activity of Anti-Inflammatory Lipopolysaccharides From Gut Microbiome. Front Pharmacol. 2020; 11:554. 10.3389/fphar.2020.00554 32425790 PMC7212368

[48] Jiang H, Deng S, Zhang J, Chen J, Li B, Zhu W, Zhang M, Zhang C, Meng Z. Acupuncture treatment for post-stroke depression: Intestinal microbiota and its role. Front Neurosci. 2023; 17:1146946. 10.3389/fnins.2023.1146946 37025378 PMC10070763

[49] Cheng Y, He CY, Tian DY, Chen SH, Ren JR, Sun HL, Xu MY, Tan CR, Fan DY, Jian JM, Sun PY, Zeng GH, Shen YY, et al. Physiological β-amyloid clearance by the liver and its therapeutic potential for Alzheimer’s disease. Acta Neuropathol. 2023; 145:717–31. 10.1007/s00401-023-02559-z 36964213

[50] Jian Y, Yuan S, Yang J, Lei Y, Li X, Liu W. Aerobic Exercise Alleviates Abnormal Autophagy in Brain Cells of APP/PS1 Mice by Upregulating AdipoR1 Levels. Int J Mol Sci. 2022; 23:9921. 10.3390/ijms23179921 36077318 PMC9456508

